# Vestibular dysfunction: a frequent problem for adults with mitochondrial disease

**DOI:** 10.1136/jnnp-2018-319267

**Published:** 2018-11-26

**Authors:** Sarah Holmes, Amanda J Male, Gita Ramdharry, Cathy Woodward, Natalie James, Iwona Skorupinska, Mariola Skorupinska, Louise Germain, Damian Kozyra, Enrico Bugiardini, Olivia V Poole, Ros Quinlivan, Michael G Hanna, Diego Kaski, Robert D S Pitceathly

**Affiliations:** 1 MRC Centre for Neuromuscular Diseases, National Hospital for Neurology and Neurosurgery, London, UK; 2 Therapy Services, National Hospital for Neurology and Neurosurgery, London, UK; 3 Neurogenetics Unit, National Hospital for Neurology and Neurosurgery, London, UK; 4 Department of Neuromuscular Diseases, UCL Queen Square Institute of Neurology, London, UK; 5 Dubowitz Neuromuscular Centre, Great Ormond Street Hospital, London, UK; 6 Department of Neuro-Otology, University College London Hospitals, London, UK

**Keywords:** neurootology, vertigo, rehabilitation

## Introduction

Mitochondrial diseases are a diverse group of genetic disorders caused by mutations in nuclear-encoded and mitochondrial DNA (mtDNA)-encoded genes. Disturbance in balance is common in adults with mitochondrial disease and usually attributed to an underlying peripheral neuropathy, skeletal myopathy and/or cerebellar disease, with limited treatment options. The vestibular system is central to balance control, and vestibular dysfunction causes vertigo, dizziness, oscillopsia, unsteadiness, and associated falls. Reports of vestibular dysfunction in mitochondrial disease are confined to small patient cohorts with limited and/or inconsistent use of neuro-otological assessments.^1–3^ Furthermore, despite the well-recognised association with hearing loss,^2^ the frequency and underlying pathophysiological basis of vestibular dysfunction in mitochondrial disease is currently unknown.

We have undertaken a large-scale, cross-sectional cohort study investigating the prevalence, classification and predictors of vestibular dysfunction in adults with mitochondrial disease.

## Patients and methods

All procedures were conducted as part of routine clinical care. The study was performed under the ethical guidelines issued by our institution, with written informed consent obtained from all participants for genetic studies. We included adults (aged 16 years or older) attending our specialist clinic between May 2016 and August 2017 with clinicopathologically and/or genetically confirmed mitochondrial disease and a suspected balance disorder. This cohort was subsequently evaluated by a specialist neuromuscular physiotherapist (SH) to exclude non-vestibular balance disorders. Patients with suspected vestibulopathy were subsequently referred for neuro-otological investigations (online supplementary table 1). Descriptive analyses were conducted on patient demographics, reported symptoms, neuro-otological and mitochondrial diagnoses, Newcastle Mitochondrial Disease Scale for Adults (NMDAS; a semiquantitative clinical rating scale for mitochondrial disease aimed at providing a validated and reproducible measurement of disease progression) total scores, hearing observations and mtDNA mutation load. NMDAS vision, hearing and balance-related subdomain scores were presented as median and IQR for those with and without neuro-otological diagnoses and for patients harbouring the m.3243A>G mutation in *MT-TL1*. The predictive value of patient-reported symptoms and hearing loss was investigated using Pearson χ² analyses and risk ratios (±95% CIs). A univariate logistic regression analysis was performed to investigate whether total NMDAS or NMDAS subdomain scores could predict neuro-otological diagnoses (peripheral or central). A Mann-Whitney test compared NMDAS scores in individuals with and without a neuro-otological diagnosis. Spearman’s correlation testing explored relationships between m.3243A>G mutant load and NMDAS total score. Statistical significance was set at p<0.05.

10.1136/jnnp-2018-319267.supp1 Supplementary data

## Results

One hundred and fourteen patients with clinicopathologically and/or genetically confirmed mitochondrial disease were evaluated during the study period. Of these, 35% (40/114) with a suspected balance disorder were referred for a physiotherapy assessment (table 1). This revealed that patient-reported symptoms clustered into five major domains: (1) dizziness; (2) light-headedness; (3) loss of balance; (4) unsteadiness and (5) falls. Five patients were diagnosed with a pure cerebellar syndrome and two with a biomechanical cause for falls. The remaining patients underwent neuro-otological investigations and a vestibular abnormality was detected in 91% (30/33), of which 77% (23/30) had peripheral vestibulopathy (table 1 and online supplementary table 1).

**Table 1:** Clinical, genetic and neuro-otological assessment findings in adults with mitochondrial disease and a suspected balance disorder

Clinical phenotype	Genetic diagnosis	Gender	Age	NMDAS score	Mitochondrial DNA mutant load			Primary neuro-otological diagnosis	Additional neuro-otological diagnoses	Patient-reported symptoms					SNHL
					Blood	Urine	Muscle			Dizziness	Light-headed	Loss of balance	Unsteadiness	Falls	
MIDD	m.3243A>G *MT-TL1*	F	70	29		67		Peripheral vestibulopathy (bilateral)				Y	Y		Bilateral
MIDD	m.3243A>G *MT-TL1*	F	33	14.5	27			Peripheral vestibulopathy (unilateral)	Vestibular migraine (central)	Y		Y		Y	Bilateral
MIDD	m.3243A>G *MT-TL1*	F	57	17.4	13	42		Peripheral vestibulopathy (unilateral)				Y	Y		Bilateral
MIDD	m.3243A>G *MT-TL1*	F	69	24.36	6	23		Peripheral vestibulopathy (unilateral)		Y	Y	Y	Y	Y	Bilateral
MIDD	m.3243A>G *MT-TL1*	F	55	51.3	No sample			BPPV		Y	Y	Y			Bilateral
MIDD	m.3243A>G *MT-TL1*	F	60	35.4	19	45		Peripheral vestibulopathy (bilateral)		Y	Y	Y	Y		Bilateral
MELAS	m.3243A>G *MT-TL1*	F	46	12.76	23	71		Peripheral vestibulopathy (bilateral)				Y			Bilateral
MIDD	m.3243A>G *MT-TL1*	F	42	23.6	21			Peripheral vestibulopathy (unilateral)	BPPV	Y	Y	Y			Bilateral
MIDD	m.3243A>G *MT-TL1*	F	50	16.1	22	62		Peripheral vestibulopathy (bilateral)				Y			Bilateral
MIDD	m.3243A>G *MT-TL1*	F	45	30.1	20			Vestibular migraine (central)		Y	Y	Y	Y		Bilateral
SNHL, RP, ataxia	m.3243A>G *MT-TL1*	F	65	27.84	14	63		Peripheral vestibulopathy (unilateral)	Vestibular migraine (central)	Y	Y	Y	Y		Bilateral
SNHL	m.3243A>G *MT-TL1*	F	47	1.16	13			Peripheral (unilateral)				Y			Bilateral
MIDD	m.3243A>G *MT-TL1*	F	40	42.92	10			Peripheral vestibulopathy (unilateral)		Y	Y	Y	Y	Y	Bilateral
SNHL, RP, HCM	m.3243A>G *MT-TL1*	F	70	13.92	13			BPPV		Y	Y	Y	Y		Bilateral
SNHL	m.3243A>G *MT-TL1*	M	50	6.2	22			Peripheral vestibulopathy (bilateral)					Y		Bilateral
MIDD	m.3243A>G *MT-TL1*	M	73	20.4	14	72		Normal				Y	Y		Bilateral
MERRF	m.8344A>G *MT-TK*	F	43	14.5	60		97	Peripheral vestibulopathy (bilateral)	BPPV	Y		Y		Y	Right
MERRF	m.8344A>G *MT-TK*	M	58	24.36	No sample			Not investigated (cerebellar)				Y	Y	Y	Normal
MERRF	m.8344A>G *MT-TK*	F	43	7.8	No sample			Not investigated (cerebellar)					Y	Y	Bilateral
MERRF	m.8344A>G *MT-TK*	F	55	31.31	75			Not investigated (cerebellar)				Y	Y	Y	Normal
MERRF	m.8344A>G *MT-TK*	F	50	25.52	No			Not investigated (cerebellar)				Y	Y	Y	Bilateral
MERRF	m.8344A>G *MT-TK*	F	69	60.32	60	74		Peripheral vestibulopathy (bilateral)	Vestibulo-cerebellar (central)	Y	Y	Y	Y	Y	Normal
RP, SNHL, DM	m.12258C>A *MT-TS2*	F	50	18.56	30			Peripheral vestibulopathy (bilateral)		Y		Y	Y		Bilateral
Multisystem, SNHL	m.8782G>A *MT-ATP6*	M	37	20.88	31	53		Not investigated (cerebellar)					Y		Bilateral
Ataxia, neuropathy	m.9176T>C *MT-ATP6*	M	29	27.84	100			Cerebellar (central)				Y	Y	Y	Bilateral
HCM, SNHL, ataxia	m.1555A>G *MT-RNR1*	M	62	19.72	100			Cerebellar (central)	BPPV			Y	Y		Left
RP, SNHL	m.10038G>A *MT-TG*	F	42	26.88	15	40	92	Peripheral vestibulopathy (bilateral)		Y	Y	Y	Y	Y	Bilateral
Leigh syndrome	m.13094T>C *MT* *-* *ND5*	M	24	20.88	38		61	Cerebellar (central)				Y	Y		Normal
CPEO, ataxia	Multiple mtDNA deletions	F	57	40.6	N/A			Peripheral vestibulopathy (unilateral)		Y	Y	Y	Y		Normal
Multisystem, SNHL	Multiple mtDNA deletions	F	64	76.56	N/A			Normal		Y	Y	Y	Y	Y	Bilateral
Multisystem, SNHL	Multiple mtDNA deletions	M	64	19.7	N/A			Peripheral vestibulopathy (bilateral)		Y		Y	Y	Y	Bilateral
CPEO, SNHL	Multiple mtDNA deletions	M	27	36.5	N/A			Peripheral vestibulopathy (bilateral)		Y	Y	Y			Right
CPEO	Single mtDNA deletion	M	63	16.24	N/A			Not investigated (biomechanical)						Y	Normal
CPEO	Single mtDNA deletion	F	26	16.24	N/A			Vestibular migraine (central)				Y		Y	Normal
CPEO, ataxia	*POLG*	M	60	31.32	N/A			Peripheral vestibulopathy (bilateral)		Y	Y	Y	Y		Bilateral
SNHL, EI	*COX10*	F	42	13.92	N/A			Not investigated (biomechanical)					Y		Bilateral
CPEO	Clinicopathological	M	44	9.28	N/A			Peripheral vestibulopathy (unilateral)	Vestibular migraine (central)	Y		Y	Y	Y	Normal
SNHL, migraine, EI	Clinicopathological	M	45	11.6	N/A			Peripheral vestibulopathy (unilateral)		Y				Y	Bilateral
Multisystem	Clinicopathological	F	32	29	N/A			Normal		Y		Y	Y		Normal
SNHL, DM, ptosis	Clinicopathological	F	72	29	N/A			Peripheral vestibulopathy (unilateral)		Y	Y	Y	Y	Y	Bilateral

Mitochondrial DNA (mtDNA) mutant load describes the percentage of mutant mtDNA in the tissue analysed. Higher scores using the Newcastle Mitochondrial Disease Scale for Adults (NMDAS) indicates greater disease burden.

BPPV, benign paroxysmal positional vertigo; COX, cytochrome c oxidase; CPEO, chronic progressive external ophthalmoplegia; DM, diabetes mellitus; EI, exercise intolerance; F, female; HCM, hypertrophic cardiomyopathy; M, male; MELAS, mitochondrial encephalopathy lactic acidosis and stroke-like episodes; MERRF, myoclonic epilepsy and red ragged fibres; MIDD, maternally inherited diabetes and deafness; RP, retinitis pigmentosa; SNHL, sensorineural hearing loss; Y, yes.

Eighty-two per cent (27/33) of patients who received neuro-otological testing had sensorineural hearing loss (SNHL), with coexistent peripheral vestibulopathy in 74% (20/27). The hearing loss was bilateral in the majority (18/20). However, bilateral peripheral vestibulopathy was present in only 55% (11/20).

An increased risk of vestibular dysfunction was identified in patients reporting symptoms of dizziness (risk ratio (RR) 3.33), light-headedness (RR 4.67) and loss of balance (RR 1.56), while hearing loss predicted peripheral vestibulopathy (RR 2.22, online supplementary table 2). Mean NMDAS total score for the cohort was 24.88 (range 1.16–76.56). No significant difference was observed in NMDAS scores between participants with and without a neuro-otological diagnosis (Z=0.27, p=0.81) and NMDAS total and subscores were not predictive of a neuro-otological diagnosis (online supplementary table 3 and online supplementary table 4).

10.1136/jnnp-2018-319267.supp2 Supplementary data

10.1136/jnnp-2018-319267.supp3 Supplementary data

10.1136/jnnp-2018-319267.supp4 Supplementary data

## Discussion

We present the first large-scale cohort study of vestibular dysfunction in adults with mitochondrial disease. The overall minimum prevalence of vestibular pathology in our symptomatic cohort of adults with mitochondrial disease was 26% (30/114), of which 77% was diagnosed as peripheral vestibulopathy. Given the known predominance of mtDNA encoding gene mutations accounting for mitochondrial disease in adults, reflected in the genetic spectrum of our cohort, we suggest these findings should be primarily applied to mtDNA-related disorders.

SNHL and peripheral vestibulopathy coexisted in 74% (20/27) of patients. A substantial proportion of this group harboured the m.3243A>G mutation (60%, 12/20), frequently associated with hearing impairment.^2^ Although bilateral SNHL was present in 78% (18/23) of patients with peripheral vestibulopathy, only 50% (9/18) had bilateral peripheral vestibulopathy. Furthermore, SNHL was present in seven patients without peripheral vestibulopathy. Taken together, these findings suggest SNHL and peripheral vestibulopathy progress at variable rates or have divergent pathophysiological mechanisms. Two patients with bilateral peripheral vestibulopathy had previously been diagnosed with unilateral peripheral vestibulopathy, suggesting a progressive decline in vestibular function. This is the first description of either bilateral or progressive vestibulopathy in patients with maternally inherited diabetes and deafness due to the m.3243A>G mutation. Benign paroxysmal positional vertigo (BPPV) was an additional neuro-otological finding in three patients. BPPV is often overlooked in patients with neurological comorbidities^4^ and is a treatable, often curable, balance disorder.

The results of this study have informed the development of a framework to support the identification, investigation and management of balance disorders in adults with mitochondrial disease attending our specialist clinic (online supplementary figure 1). Prospective analysis will further validate the sensitivity and specificity of this algorithm and may have important implications for other complex neurological disorders associated with vestibular dysfunction.

10.1136/jnnp-2018-319267.supp5 Supplementary data

This study confirms that vestibular dysfunction is an important manifestation of mitochondrial disease in adults. Given the clear benefits of vestibular rehabilitation,^5^ identifying and diagnosing people with vestibular dysfunction is important to ensure appropriate and effective management of these disabling symptoms. Consequently, referral for neuro-otological assessment should be considered in all adults with mitochondrial disease reporting dizziness, unsteadiness or SNHL. Ongoing longitudinal analysis of our cohort is currently underway to determine the natural history of vestibular dysfunction in adults with mitochondrial disease.
