# Supplementary material for: Vestibular dysfunction: a frequent problem for adults with mitochondrial disease
Source: J Neurol Neurosurg Psychiatry. 2018 Nov 26;90(7):838–41. doi: 10.1136/jnnp-2018-319267 (PMC6585572; doi:10.1136/jnnp-2018-319267)
Supplement: Supplementary data [file jnnp-2018-319267supp001.docx]

**Supplementary Table 1:** Neuro-otological tests completed in adults with mitochondrial disease and a suspected balance disorder

| **Clinical phenotype** | **Genetic diagnosis** | **Gender** | **Age** | **NMDAS score** | **Mitochondrial DNA mutant load** | | | **Primary**  **neuro-otological diagnosis** | **Additional**  **neuro-otological diagnoses** | **Neuro-otological investigations** | | | | | |
| --- | --- | --- | --- | --- | --- | --- | --- | --- | --- | --- | --- | --- | --- | --- | --- |
|  |  |  |  |  | **Blood** | **Urine** | **Muscle** |  |  | **PTA** | **Tympanometry** | **Head impulse test** | **Caloric** | **Oculography** | **cVEMPS** |
| MIDD | m.3243A>G *MT-TL1* | F | 70 | 29 |  | 67 |  | Peripheral vestibulopathy (bilateral) |  | Y | Y | Y | Y | Y | N |
| MIDD | m.3243A>G *MT-TL1* | F | 33 | 14.5 | 27 |  |  | Peripheral vestibulopathy (unilateral) | Vestibular migraine (central) | Y | Y | Y | Y | Y | N |
| MIDD | m.3243A>G *MT-TL1* | F | 57 | 17.4 | 13 | 42 |  | Peripheral vestibulopathy (unilateral) |  | Y | Y | Y | N | N | N |
| MIDD | m.3243A>G *MT-TL1* | F | 69 | 24.36 | 6 | 23 |  | Peripheral vestibulopathy (unilateral) |  | Y | Y | N | Y | Y | N |
| MIDD | m.3243A>G *MT-TL1* | F | 55 | 51.3 | No sample | | | BPPV |  | Y | Y | Not available | | | |
| MIDD | m.3243A>G *MT-TL1* | F | 60 | 35.4 | 19 | 45 |  | Peripheral vestibulopathy (bilateral) |  | Y | Y | Y | N | N | Y |
| MELAS | m.3243A>G *MT-TL1* | F | 46 | 12.76 | 23 | 71 |  | Peripheral vestibulopathy (bilateral) |  | Y | Y | Y | Y | Y | Y |
| MIDD | m.3243A>G *MT-TL1* | F | 42 | 23.6 | 21 |  |  | Peripheral vestibulopathy (unilateral) | BPPV | Y | Y | Y | Y | Y | N |
| MIDD | m.3243A>G *MT-TL1* | F | 50 | 16.1 | 22 | 62 |  | Peripheral vestibulopathy (bilateral) |  | Y | Y | Y | Y | N | N |
| MIDD | m.3243A>G *MT-TL1* | F | 45 | 30.1 | 20 |  |  | Vestibular migraine (central) |  | Y | Y | Y | N | Y | N |
| SNHL, RP, ataxia | m.3243A>G *MT-TL1* | F | 65 | 27.84 | 14 | 63 |  | Peripheral vestibulopathy (unilateral) | Vestibular migraine (central) | Y | Y | Y | Y | N | N |
| SNHL | m.3243A>G *MT-TL1* | F | 47 | 1.16 | 13 |  |  | Peripheral vestibulopathy (unilateral) |  | Y | Y | Y | Y | Y | Declined |
| MIDD | m.3243A>G *MT-TL1* | F | 40 | 42.92 | 10 |  |  | Peripheral vestibulopathy (unilateral) |  | Y | Y | Y | Y | N | Y |
| SNHL, RP, HCM | m.3243A>G *MT-TL1* | F | 70 | 13.92 | 13 |  |  | BPPV |  | Y | Y | Y | Y | Y | Y |
| SNHL | m.3243A>G *MT-TL1* | M | 50 | 6.2 | 22 |  |  | Peripheral vestibulopathy (bilateral) |  | Y | Y | Y | N | Y | Y |
| MIDD | m.3243A>G *MT-TL1* | M | 73 | 20.4 | 14 | 72 |  | Normal |  | Y | Y | Y | N | Y | N |
| MERRF | m.8344A>G *MT-TK* | F | 43 | 14.5 | 60 |  | 97 | Peripheral vestibulopathy (bilateral) | BPPV | Y | Y | Y | Y | Y | N |
| MERRF | m.8344A>G *MT-TK* | M | 58 | 24.36 | No sample | | | Not investigated (cerebellar) | | | | | | | |
| MERRF | m.8344A>G *MT-TK* | F | 43 | 7.8 | No sample | | | Not investigated (cerebellar) | | | | | | | |
| MERRF | m.8344A>G *MT-TK* | F | 55 | 31.31 | 75 |  |  | Not investigated (cerebellar) | | | | | | | |
| MERRF | m.8344A>G *MT-TK* | F | 50 | 25.52 | No |  |  | Not investigated (cerebellar) | | | | | | | |
| MERRF | m.8344A>G *MT-TK* | F | 69 | 60.32 | 60 | 74 |  | Peripheral vestibulopathy (bilateral) | Vestibulo-cerebellar (central) | Y | Y | Y | N | Y | N |
| RP, SNHL, DM | m.12258C>A *MT-TS2* | F | 50 | 18.56 | 30 |  |  | Peripheral vestibulopathy (bilateral) |  | Y | Y | Y | N | Y | N |
| Multisystem, SNHL | m.8782G>A *MT-ATP6* | M | 37 | 20.88 | 31 | 53 |  | Not investigated (cerebellar) | | | | | | | |
| Ataxia, neuropathy | m.9176T>C *MT-ATP6* | M | 29 | 27.84 | 100 |  |  | Cerebellar (central) |  | Y | Y | Y | N | Y | N |
| HCM, SNHL, ataxia | m.1555A>G *MT-RNR1* | M | 62 | 19.72 | 100 |  |  | Cerebellar (central) | BPPV | Y | Y | Y | Y | Y | N |
| RP, SNHL | m.10038G>A *MT-TG* | F | 42 | 26.88 | 15 | 40 | 92 | Peripheral vestibulopathy (bilateral) |  | Y | Y | Y | Y | Y | Y |
| Leigh syndrome | m.13094T>C *MT-ND5* | M | 24 | 20.88 | 38 |  | 61 | Cerebellar (central) |  | Y | Y | Y | N | Y | Y |
| N/A | Multiple mtDNA deletions | F | 57 | 40.6 | N/A | | | Peripheral vestibulopathy (unilateral) |  | Y | Y | N/A (CPEO) | Y | N/A (CPEO) | N/A (CPEO) |
| Multisystem, SNHL | Multiple mtDNA deletions | F | 64 | 76.56 | N/A | | | Normal |  | Y | Y | Y | Y | Y | Y |
| Multisystem, SNHL | Multiple mtDNA deletions | M | 64 | 19.7 | N/A | | | Peripheral vestibulopathy (bilateral) |  | Y | Y | Y | Y | Y | Y |
| CPEO, SNHL | Multiple mtDNA deletions | M | 27 | 36.5 | N/A | | | Peripheral vestibulopathy (bilateral) |  | Y | Y | N/A (CPEO) | Y | Y | Y |
| CPEO | Single mtDNA deletion | M | 63 | 16.24 | N/A | | | Not investigated (biomechanical) | | | | | | | |
| CPEO | Single mtDNA deletion | F | 26 | 16.24 | N/A | | | Vestibular migraine (central) |  | Y | Y | Y | Y | Y | Y |
| CPEO, ataxia | *POLG* | M | 60 | 31.32 | N/A | | | Peripheral vestibulopathy (bilateral) |  | Y | Y | Y | Y | Y | Y |
| SNHL, EI | *COX10* | F | 42 | 13.92 | N/A | | | Not investigated (biomechanical) | | | | | | | |
| CPEO | Clinicopathological | M | 44 | 9.28 | N/A | | | Peripheral vestibulopathy (unilateral) | Vestibular migraine (central) | Y | Y | N | Y | Y | N |
| SNHL, migraine, EI | Clinicopathological | M | 45 | 11.6 | N/A | | | Peripheral vestibulopathy (unilateral) |  | Y | Y | Y | N | Y | Y |
| Multisystem | Clinicopathological | F | 32 | 29 | N/A | | | Normal |  | Y | Y | Y | N | N | N |
| SNHL, DM, ptosis | Clinicopathological | F | 72 | 29 | N/A | | | Peripheral vestibulopathy (unilateral) |  | Y | Y | Y | Y | N | Y |

Mitochondrial DNA (mtDNA) mutant load describes the percentage of mutant mtDNA in the tissue analysed. Higher scores using the Newcastle Mitochondrial Disease Scale for Adults (NMDAS) indicates greater disease burden. Abbreviations: BPPV, benign paroxysmal positional vertigo; COX, cytochrome *c* oxidase; CPEO, chronic progressive external ophthalmoplegia; cVEMP, cervical vestibular evoked myogenic potential; DM, diabetes mellitus; EI, exercise intolerance; F, female; HCM, hypertrophic cardiomyopathy; M, male; MELAS, mitochondrial encephalopathy lactic acidosis and stroke-like episodes; MERRF, myoclonic epilepsy and red ragged fibres; MIDD, maternally inherited diabetes and deafness; N/A, not applicable; N, no; PTA, pure tone audiogram; RP, retinitis pigmentosa; SNHL, sensorineural hearing loss; Y, yes.
