# Supplementary material for: Vestibular dysfunction: a frequent problem for adults with mitochondrial disease
Source: J Neurol Neurosurg Psychiatry. 2018 Nov 26;90(7):838–41. doi: 10.1136/jnnp-2018-319267 (PMC6585572; doi:10.1136/jnnp-2018-319267)
Supplement: Supplementary data [file jnnp-2018-319267supp002.docx]

**Supplementary Table 2:** Risk ratio and Chi squared results for hearing loss and diagnosis of peripheral vestibular disorder in adults with mitochondrial disease

| **Presence of symptom** | **Risk of PVD**  **(Risk Ratio 95% CI)** | **Chi Squared** | **P Value** |
| --- | --- | --- | --- |
| Hearing loss | 2.22 (0.83-5.92) | 4.13 | 0.042* |

Abbreviations: CI, Confidence Intervals; PVD, Peripheral Vestibular Disorder; * = p≤0.05.
