# Supplementary material for: Vestibular dysfunction: a frequent problem for adults with mitochondrial disease
Source: J Neurol Neurosurg Psychiatry. 2018 Nov 26;90(7):838–41. doi: 10.1136/jnnp-2018-319267 (PMC6585572; doi:10.1136/jnnp-2018-319267)
Supplement: Supplementary data [file jnnp-2018-319267supp003.docx]

**Supplementary Table 3:** Summary of NMDAS descriptive statistics and logistic regression results to ascertain prediction of neuro-otological diagnosis in adults with mitochondrial disease

| **NMDAS** | **Median (IQR)** | **R^2^** | **P Value** |
| --- | --- | --- | --- |
| Total Score | 22.82 (16.24-21.55) | 0.020 | 0.40 |
| Vision | 1 (0-2) | 0.064 | 0.31 |
| Hearing | 2 (0.3) | 0.15 | 0.055 |
| Balance | 2 (1-3) | 0.018 | 0.46 |
| Ptosis | 0 (0-1) | 0.010 | 0.57 |
| CPEO | 0 (0-0) | 0.030 | 0.37 |
| Ataxia | 2 (0-3) | 0.018 | 0.46 |
| Neuropathy | 0 (0-0.5) | 0.0030 | 0.77 |

Abbreviations: CPEO, Chronic Progressive External Ophthalmoplegia; IQR, Interquartile Range; NMDAS, Newcastle Mitochondrial Disease Scale for Adults.

.
