# Supplementary material for: Vestibular dysfunction: a frequent problem for adults with mitochondrial disease
Source: J Neurol Neurosurg Psychiatry. 2018 Nov 26;90(7):838–41. doi: 10.1136/jnnp-2018-319267 (PMC6585572; doi:10.1136/jnnp-2018-319267)
Supplement: Supplementary data [file jnnp-2018-319267supp004.docx]

**Supplementary Table 4:** Balance related NMDAS subdomain median and interquartile ranges in adults with mitochondrial disease with and without a neuro-otological diagnosis, and for those harbouring the m.3243A>G mutation in *MT-TL1*

| **Groups** |  | **Vision** | **Hearing** | **Gait instability** | **Migraine headaches** | **Ptosis** | **CPEO** | **Myopathy** | **Ataxia** | **Neuropathy** |
| --- | --- | --- | --- | --- | --- | --- | --- | --- | --- | --- |
| No neuro-otological diagnosis | Median | 2 | 2 | 2 | 2 | 3 | 3 | 1 | 3 | 2 |
|  | IQR | 0.5 | 1 | 2 | 0 | 0 | 0 | 1 | 2 | 0 |
| Confirmed neuro-otological diagnosis | Median | 2 | 3 | 2 | 2 | 1 | 0 | 1 | 3 | 2 |
|  | IQR | 1 | 2 | 2 | 0 | 0 | 0 | 1 | 2 | 0 |
| m.3243A>G mutation | Median | 0.5 | 3 | 1.5 | 0 | 0 | 0 | 0 | 2 | 0 |
|  | IQR | 1 | 1 | 2 | 1 | 0.25 | 0 | 1 | 3 | 0.25 |

Abbreviations: CPEO, Chronic Progressive External Ophthalmoplegia; IQR, Interquartile Range; NMDAS, Newcastle Mitochondrial Disease Scale for Adults.
