# Supplementary material for: Vestibular dysfunction: a frequent problem for adults with mitochondrial disease
Source: J Neurol Neurosurg Psychiatry. 2018 Nov 26;90(7):838–41. doi: 10.1136/jnnp-2018-319267 (PMC6585572; doi:10.1136/jnnp-2018-319267)
Supplement: Supplementary data [file jnnp-2018-319267supp005.docx]

**Supplementary Figure 2:** Framework to support the identification, investigation and management of suspected balance disorders in adults with mitochondrial disease

- If positive head thrust test, refer for neuro-otological investigations
- Refer for vestibular rehabilitation
- Manage migraine
- Refer for vestibular rehabilitation (as required)
- Positional tests and repositioning manoeuvres
- Refer for vestibular rehabilitation (as required)
- Refer for neuro-otological investigations
- Refer for vestibular rehabilitation

**Management:**

**Potential cause of balance disorder:**

Peripheral vestibular disorder

Migraine

Visual dependence

BPPV

- Dizziness or blurry vision when moving head or body?
- Bobbing vision when walking?
- Imbalance?
- Head or neck pain?
- Visual aura?
- Light/sound sensitivity?
- Nausea?
- Dizzy on turning over in bed?
- Dizzy/loss of balance bending or moving head down or up?
- Imbalance in the dark?
- Dizzy or imbalance in crowds, escalators, supermarkets?

**Patient reported symptoms:**

Dizziness, light-headedness, loss of balance, unsteadiness, falls, hearing loss

**Additional questions:**
